# Folic-Acid-Conjugated Poly (Lactic-Co-Glycolic Acid) Nanoparticles Loaded with Gallic Acid Induce Glioblastoma Cell Death by Reactive-Oxygen-Species-Induced Stress

**DOI:** 10.3390/polym16152161

**Published:** 2024-07-30

**Authors:** Maria João Ramalho, Bruna Alves, Stéphanie Andrade, Jorge Lima, Joana Angélica Loureiro, Maria Carmo Pereira

**Affiliations:** 1 LEPABE—Laboratory for Process Engineering, Environment, Biotechnology and Energy, Faculty of Engineering, University of Porto, Rua Dr. Roberto Frias, 4200-465 Porto, Portugalstephanie@fe.up.pt (S.A.); jasl@fe.up.pt (J.A.L.); 2 ALiCE—Associate Laboratory in Chemical Engineering, Faculty of Engineering, University of Porto, Rua Dr. Roberto Frias, 4200-465 Porto, Portugal; 3 i3S—Instituto de Investigação e Inovação em Saúde, Universidade do Porto, R. Alfredo Allen, 4200-135 Porto, Portugal; jlima@i3s.up.pt; 4 Ipatimup—Instituto de Patologia e Imunologia Molecular da Universidade do Porto, Rua Júlio Amaral de Carvalho 45, 4200-135 Porto, Portugal; 5 Faculty of Medicine, University of Porto, Alameda Prof. Hernâni Monteiro, 4200-319 Porto, Portugal

**Keywords:** nanomedicine, brain tumor, natural compound, oxidative stress, drug toxicity, central composite design

## Abstract

Glioblastoma (GBM) conventional treatment is not curative, and it is associated with severe toxicity. Thus, natural compounds with anti-cancer properties and lower systemic toxicity, such as gallic acid (GA), have been explored as alternatives. However, GA’s therapeutic effects are limited due to its rapid metabolism, low bioavailability, and low permeability across the blood–brain barrier (BBB). This work aimed to develop poly (lactic-co-glycolic acid) (PLGA) nanoparticles (NPs) modified with folic acid (FA), as its receptor is overexpressed in BBB and GBM cells, for GA delivery to enhance its therapeutic efficacy. The preparation of NPs was optimized by a central composite design (CCD). The obtained NPs showed physicochemical features suitable for drug internalization in BBB and tumor cells (sizes below 200 nm, monodispersity, and negative surface charge) and the ability to maintain a slow and sustained release for 40 days. In vitro studies using a human GBM cell line (U215) revealed the NPs’ ability to accumulate in the target cells, further promoting GA antiproliferative activity by inducing the production of intracellular reactive oxygen species (ROS). Furthermore, GA encapsulation in the developed nanosystems conferred higher protection to healthy cells.

## 1. Introduction

Glioblastoma (GBM) is part of the family of malignant gliomas, being highly proliferative, aggressive, and invasive. The standard treatment of this malignancy, known as the Stupp protocol, involves surgical resection, followed by radiotherapy combined with adjuvant chemotherapy with the alkylating agent temozolomide (TMZ). However, although this multimodal approach can prolong the survival time of patients to about 12–18 months, is not curative [1]. Thus, this type of tumor has a reduced survival rate, with a high probability of recurrence. Moreover, the chemotherapeutic drugs used in treating GBM are associated with severe toxicity that compromises their use [2]. The most common side effects are nausea, vomiting, skin rashes, intracranial edema and infection, cerebrospinal fluid leak, myelosuppression, and pulmonary fibrosis [3].

Hence, natural compounds with proven anti-cancer activity have emerged as alternative approaches due to their lower toxicity for healthy tissues [4]. Between 1980 and 2019, the percentage of new drugs derived from natural products in combating cancer was approximately 25% [5]. Regarding GBM, different natural compounds have been explored such as curcumin [6], cardamom [7], resveratrol [8], and gallic acid (GA). 

GA, 3,4,5-trihydroxy benzoic acid, is a natural polyhydroxyphenolic compound that is found in various foods and plants such as nuts, hazelnuts, cloves, tea leaves, strawberries, and others [9,10]. GA exhibits several biological properties: anti-bacterial, anti-viral, antioxidant, and anti-carcinogenic properties [11]. The anti-carcinogenic activity of GA has been demonstrated in several in vitro and in vivo studies for GBM [12] for different types of cancer, including lung, prostate, breast, leukemia, and others [10,13], without significant toxicity towards healthy cells [10,14].

However, as for other natural compounds, the systemic administration of GA faces several limitations. Like most natural compounds, GA has low solubility and stability, which decreases its bioavailability in the target tissues, compromising its use [15]. In addition, the anatomical location of this malignant tumor implies the passage of the therapeutic agents through the blood–brain barrier (BBB), which is difficult since this physiological barrier blocks the passage of most compounds. In this sense, it is essential to develop alternative approaches to deliver natural compounds, such as using nanoparticles (NPs) with brain-targeting ability [16]. 

Although using NPs for brain delivery offers significant advantages, the nanoencapsulation of GA for GBM therapy has been little explored so far. In fact, until this date, only two works have been published reporting the encapsulation of GA in NPs to be used for GBM application. In both published studies, the authors developed metallic NPs (gold [17] and iron NPs [18]). However, the administration of metallic NPs has some limitations for health purposes, such as their non-degradability and associated toxicity. 

Thus, this work proposes the encapsulation of GA in poly (lactic-co-glycolic acid) (PLGA) NPs. This polymer was selected for NP production due to being FDA-approved, biodegradable, and biocompatible [19]. Several studies have demonstrated the advantages of PLGA NPs for drug delivery, demonstrating the versatility of this polymer in encapsulating a wide range of drugs in particular for application in GBM [20,21,22,23]. When compared to other delivery systems, PLGA NPs exhibit some advantages. For example, liposomes and micelles, while effective in certain applications, often suffer from stability issues and can be rapidly cleared from the bloodstream [24]. Furthermore, the degradation rate of PLGA can be tailored by adjusting the ratio of lactic to glycolic acid, allowing for controlled release over a desired period. Additionally, the possibility of modifying the surface of PLGA NPs allows for targeted delivery, enhancing therapeutic efficacy and reducing side effects [25].

After production, the NPs were optimized by implementing a central composite design (CCD). The NPs were functionalized with folic acid (FA) to improve their brain-tumor-targeting ability, which will increase the NPs’ specificity and consequently their therapeutic efficacy. The effect of FA functionalization in the in vitro uptake of the developed NPs was evaluated in human GBM cells (U251) and healthy immortalized astrocytes (NHA). Then, the same cell lines were employed to evaluate the antiproliferative activity of the developed FA-GA-NPs as the entrapped GA’s ability to induce reactive oxygen species (ROS) production. 

## 2. Materials and Methods

### 2.1. Materials

Polyvinyl alcohol 4–88 (Mowiol^®^ 4–88, MW 31,000) (PVA), PLGA Resomer^®^ RG 503 H (50:50; MW 24,000–38,000), 1-ethyl-3-(3-dimethylaminopropyl) carbodiimide (EDC) (MW 191.70), folic acid (FA) (≥97%, MW 441.40), gallic acid (GA) (97.5–102.5%, MW 170.12), phosphate-buffered saline (PBS), acetic acid (≥98%, MW 60.05), coumarin-6 (99%, MW 350.43), NaOH (1.0 M, MW 40.0), *tert*-Butyl hydroperoxide solution (5.0–6.0 M in decane MW 90.12), dichloro-dihydro-fluorescein diacetate (DCFH-DA, 97%, MW487.29), Tris(hydroxymethyl)aminomethane (≥99.8%, MW 141.14), Sulforhodamine B (SRB) (MW 580.66), and trichloroacetic acid (TCA) (99%, MW 163.38) were obtained from Sigma Aldrich (St. Louis, MO, USA). Ethyl acetate (EA) (99.6%, ACS reagent, MW 88.11) was acquired from BioVision (Waltham, MA, EUA). Trypsin (Gibco™ TrypLE™) and fetal bovine serum (Gibco™ FBS) were supplied by Fisher Scientific (Hampton, NH, USA). High-glucose Dulbecco’s Modified Eagle Medium (DMEM) was purchased from Capricorn Scientific (Ebsdorfergrund, Germany). Penicillin–streptomycin solution (×100) and funzigone were purchased from Biowest LCC (Riverside, MO, USA). Trypan blue (≥70%, MW 960.80) was obtained from Biochem Chemopharma (Cosne-Cours-sur-Loire, France). Triton X-100 was acquired from Fluka (Buchs, Switerzland). 

### 2.2. Cells and Cell Culture

Two different cell lines were used, a GBM tumor cell line (U251) and a healthy cell line (immortalized human astrocytes, NHA) as control. Both lines were grown in Dulbecco’s Modified Eagle Medium (DMEM) supplemented with 10% (*v*/*v*) FBS. To avoid contamination, 1% (*v*/*v*) penicillin–streptomycin and 0.5% (*v*/*v*) fungizone were added to the culture. The cells were trypsinized for cell passage to detach them from culture flasks. The cultures were maintained at 37 °C in an incubator with 5% CO_2_.

### 2.3. Optimization of the NP Preparation Protocol

#### 2.3.1. Experimental Design 

A CCD was implemented to produce optimized NPs (Design Expert software, version 11.1.2.0, Stat-Ease Inc., Minneapolis, MN, USA). The amount of PLGA, the percentage of PVA, the number of FA molecules per NP, and the sonication cycle number were chosen as the experimental parameters (independent variables) due to representing the most preponderant variables for preparing the NPs in preliminary experiments. These parameters were varied in five levels: −1, −α, +1, 0, and +α, with an α value of 1.54671 (Table 1), automatically determined by the software after choosing an orthogonal quadratic design [26]. 

Table S1 (supplementary file) shows that 30 independent formulations were prepared, including 6 replicas of the center point, to evaluate the experimental reproducibility [15]. To avoid bias, the order of the formulations was set randomly. The responses (dependent variables) under study were the size of the NPs, the polydispersity index (PDI), the zeta potential, and the encapsulation efficiency (EE) of GA. The statistical analysis was performed by analysis of variance (ANOVA), and with *p*-values lower than 0.05 being considered significant, with a 95% confidence interval. For each studied response variable, a regression equation (Equation (1)) was fitted to determine the significance between the independent variables and the obtained response.

(1) $$\begin{array}{c} Y=\;b_{0}+b_{1}X_{1}+\cdots +b_{k}X_{k}+b_{12}X_{1}X_{2}+b_{13}X_{1}X_{3}+\cdots \\ \;\;\;\;\;\;\;+\;b_{k-1,k}X_{k-1}X_{k}+\;b_{11}X_{1}^{2}+\cdots +b_{kk}X_{k}^{2}+\epsilon \; \end{array}$$

#### 2.3.2. Preparation of the GA-Loaded PLGA NPs and Conjugation with FA

GA-loaded NPs were produced using the single-emulsion evaporation technique. For that, 1.0 mg of GA and a known amount of PLGA were dissolved in ethyl acetate. The amount of PLGA was varied as described in Table S1 (supplementary file). A volume of 2.0 mL of an aqueous solution of PVA was added to the previously prepared organic solution, and the preparation was then emulsified by mechanical agitation. The amount of PVA was varied as described in Table S1 (supplementary file). Next, the emulsion was subjected to 10 s sonication cycles with an amplitude of 70% and a frequency of 24 kHz (probe sonicator, UP400S, Hielscher, Berlin, Germany). The number of sonication cycles was varied as described in Table S1 (supplementary file). The emulsion was quickly transferred to a magnetic stir plate at 800 rpm (rotations per minute) (Colosquid, ika^®^, electromagnetic stirrer) to completely remove the ethyl acetate by evaporation. The NPs were then collected by centrifugation, with an increasing centrifugation speed ranging from 5000 to 14,500 rpm. The pellet containing the NPs was resuspended in ultrapure water, and the supernatant was saved for further non-encapsulated GA quantification.

The conjugation of FA to the surface of the prepared GA-NPs was achieved through the crosslinking reaction of the terminal carboxylic group of PLGA with the primary amine of FA, mediated by 1-ethyl-3-(3-dimethylaminopropyl) carbodiimide (EDC). A molar excess (approximately 20-fold) of EDC was added to the GA-NPs and incubated at room temperature for 30 min with agitation on a stir plate (Colosquid, ika^®^, electromagnetic stirrer). Then, a molar excess of FA was added to the suspension of NPs with the activated carboxylic group and incubated at room temperature for 1 h with mechanical shaking. The NPs were collected by centrifugation and resuspended in ultrapure water.

### 2.4. NP Physicochemical Characterization

The size distribution and zeta potential of the NPs were evaluated by Dynamic Light Scattering (DLS) and Electrophoretic Light Scattering (ELS), respectively, using a ZetaSizer Nano ZS (Malvern Instruments, Malvern, UK). The NPs were diluted in ultrapure water to a final concentration of 1 mg/mL. The samples were analyzed with a 633 nm red laser at 25 °C with an angle of 173°, and the results obtained were given by the intensity distribution. Data treatment was performed by the ZetaSizer Software (version 8, Malvern Instruments, Malvern, UK). Weekly DLS measurements were also performed for 8 weeks to evaluate the colloidal stability of the NPs in storage conditions (aqueous suspension, 4 °C). 

Attenuated Total Reflectance Fourier-Transform Infrared Spectroscopy (ATR-FTIR) was used to confirm the successful NPs’ conjugation with FA. A Bruker Alpha-P FTIR Spectrometer (Bruker Optics Inc., Billerica, MA, USA) and Opus Software (version 6.5, Bruker Optik GmbH) were used to record the FTIR spectra of the produced NPs. The spectra were attained in absorbance mode with 64 scans at a resolution of 4 cm^−1^ for both the sample and background observations, with a wavenumber range of 4000–375 cm^−1^. The acquired spectra’s intensity range was normalized and smoothed using Graphpadprism (version 9.1.2, GraphPad Software). A 10 µL drop of each sample was deposited on the crystal for examination, and it was then dried with a nitrogen flow. FTIR measurements were performed for FA stock solution, PLGA polymer, and GA-loaded PLGA NPs before and after FA conjugation.

Morphological analysis was conducted by transmission electron microscopy (TEM). A volume of 10 µL of the prepared FA-GA-loaded NPs at a concentration of 10 mg/mL was placed on copper grids (Formvar/Carbon-400 mesh Copper, Agar Scientific, Essex, UK) and negatively stained with 2% (*v*/*v*) uranyl acetate. The NPs were visualized with an accelerating voltage of 80 kV using a Jeol JEM 1400 electron microscope (Tokyo, Japan). At least six images were acquired. 

### 2.5. Determination of Encapsulation Efficiency

The EE of the produced NPs was determined indirectly by quantifying the non-encapsulated GA in the supernatant by UV–vis absorbance at the wavelength of 261 nm (microplate reader, Synergy 2 Microplate Reader, BioTek, Cheadle, UK). The obtained absorbance values were correlated to a GA calibration curve in PVA. The supernatant obtained after NPs’ functionalization with FA was also measured to quantify the GA that might be eventually lost during this step. FA at the used concentration does not exhibit absorbance signal, not interfering with GA quantification. The EE (%) values for GA were determined using the following equations:

(2) $$\begin{array}{c} Encapsulated\;GA\;\left(mg\right)\\ \;\;\;\;\;\;=total\;GA\;\left(mg\right)-(non-encapsulated\;GA\;\left(mg\right)\\ \;\;\;\;\;\;+GA\;lost\;in\;functionalization\;\left(mg\right)) \end{array}$$

(3) $$EE\;\left(\%\right)=\frac{encapsulated\;GA\;\left(mg\right)}{total\;amount\;of\;GA\;\left(mg\right)}\;100$$

### 2.6. In Vitro Release Experiments 

The in vitro release profile of GA from FA-functionalized NPs was evaluated over 40 days under simulated physiological conditions at 37 °C under gentle agitation (100 rpm). Two different experiments were performed using PBS (0.01 M) with different pH values (6.4 and 7.4) to simulate distinct physiological conditions. For both experiments, the NPs were suspended in 3 mL of PBS and then divided into 10 aliquots. Then, at predetermined timepoints, an aliquot of the NPs was centrifuged to separate the released GA from the NPs. The released GA in the supernatant was quantified by UV–vis absorbance at the wavelength of 261 nm (microplate reader, Synergy 2 Microplate Reader, BioTek, UK). DLS measurements were performed to evaluate the colloidal stability of the NPs under simulated physiological conditions for the duration of these experiments. 

### 2.7. In Vitro Cell Uptake and FA Competitive Binding Studies

NPs’ internalization was assessed by fluorescence quantification of coumarin-6-labeled NPs. Coumarin-6 was loaded into the NPs as previously reported [27]. For the experiment, U251 and NHA cells were grown in 96-well plates at a density of 8000 cells per well (TPP^®^ tissue culture plates). After 24 h to allow cell adhesion, the cells were incubated with coumarin-6-labeled NPs diluted in DMEM at a PLGA concentration of 0.5 mg/mL. To evaluate the effect of FA conjugation on the NPs’ uptake, the cells were treated with FA-conjugated or non-conjugated NPs. Also, two incubation periods (30 and 120 min) were tested to assess the influence of incubation time on the NPs’ uptake. After incubation, the non-internalized NPs were removed by washing the cells with cold PBS. To allow for fluorescence quantification, the cells were disrupted using a lysis buffer composed of 0.1% (*v*/*v*) of Triton X-100 in 0.1 M NaOH. Internalized NPs were then quantified by fluorescence measurements at excitation/emission wavelengths of 430/485 nm, respectively (Microplate reader, BioTek Synergy HT Microplate Reader-BioTek, UK). 

A competitive binding study was performed to further investigate the mechanism of NPs’ uptake. To block the folate receptor, cells were pre-treated for 1 h with excess folate diluted in DMEM at concentrations ranging from 1.0 to 1.0 × 10^5^ nM. After washing the cells with plain DMEM to remove unbound folate, the cells were treated with FA-conjugated NPs or non-conjugated NPs for 120 min. After treatment, the cells were prepared for fluorescence measurements as described above. 

### 2.8. In Vitro Cytotoxicity Studies by Sulforhodamine B Assay

To test the effect of GA-loaded NPs functionalized with FA, the sulforhodamine (SRB) colorimetric assay was used for both cell lines (U251 and NHA). Free GA and unloaded FA-PLGA NPs were also tested as controls. 

For this assay, the cells were grown in 96-well plates at a density of 1000 cells per well (TPP^®^ tissue culture plates). The cells were incubated at 37 °C for 24 h in a humidified incubator at 5% CO_2_ for cell adhesion. Then, the free drug, FA-GA-loaded NPs, and unloaded FA-PLGA NPs were diluted in DMEM medium and added to the cells. The GA concentrations used were 10–1000 µM for free GA and 10–2000 µM for the FA-GA-loaded NPs. Control NPs were added at a PLGA concentration ranging from 0.15 to 0.75 mg/mL. Untreated cells were used as negative controls. After a 72 h incubation period, the SBR protocol was subsequently performed. Briefly, the cells were incubated with 10% (*w*/*v*) TCA at 4 °C for 1 h to fix the cells. Then, the cells were stained with SRB 0.4% (*w*/*v*) in 1% (*v*/*v*) acetic acid for 20 min. To remove the non-incorporated excess dye, the cells were washed twice with 1% (*v*/*v*) acetic acid. Finally, 100 µL of Tris buffer solution (10 mM) was added to solubilize the incorporated dye and allow the consequent absorbance reading. Cell protein was quantified by UV–vis absorbance at 560 nm (BioTek Synergy HT Microplate Reader-BioTek, UK). Cell survival was determined by the following equation, where *T* represents the absorbance of the treated wells, while *C* corresponds to the absorbance values of the non-treated wells:

(4) $$Cell\;survival\;\left(\%\right)=\frac{T}{C}\times 100$$

### 2.9. In Vitro ROS Production 

The DCFH-DA probe assay was used to quantify ROS production induced by the developed FA-GA-PLGA NPs. Briefly, U251 and NHA cells were seeded in 96-well plates at a density of 2000 cells per well (TPP^®^ tissue culture plates). To allow cell adhesion, the cells were incubated at 37 °C for 24 h in a humidified incubator at 5% CO_2_. The cells were then treated with increasing concentrations of the free drug or FA-GA-loaded NPs diluted in DMEM. The GA concentrations used were 10–200 µM for free GA and 10–1000 µM for FA-GA-loaded NPs. Tert-butyl hydroperoxide (t-BHP) at a concentration of 200 µM was added as the positive control. Negative control cells were left untreated. After 72 h of incubation, DMEM containing the treatment was removed, and the cells were washed once with plain DMEM. Cells were then incubated with 20 µM DCFH-DA for 30 min, protected from light. Fluorescence was measured at excitation/emission wavelengths of 485/540 nm, respectively (Microplate reader, BioTek Synergy HT Microplate Reader-BioTek, UK). The fluorescence values were then normalized to cell number determined by the SRB cell viability assay.

### 2.10. Statistical Analysis

The results are presented as the mean ± standard deviation (SD) for at least three independent studies. Student’s *t*-test was applied for statistical analysis, with a 95% confidence interval, and *p* < 0.05 was considered significant.

## 3. Results and Discussion

### 3.1. Optimization of NPs by Experimental Design

The preparation protocol of GA-NPs functionalized with FA was optimized by using a CCD, as an alternative to the traditional technique of one factor at a time (OFAT) since, unlike the latter, it allows the optimization of the protocol by changing more than one experimental parameter at the same time [28]. The CCD provides results more precisely by constructing a second-order quadratic model capable of examining each factor in five distinct levels, allowing the estimation of the quadratic terms. This model is inserted in the response surface analysis, where the models can plot contours in more than one dimension to describe the response through quadratic or cubic equations. This way, the relationship between the different parameters is presented as polynomials with curvature and can go beyond linearity [29]. 

In preliminary experiments, four independent variables were identified and chosen as those preponderant for directly affecting the physicochemical characteristics of the NPs: mass (mg) of PLGA, percentage (%) of PVA, number of FA molecules per NP, and number of sonication cycles. The remaining experimental parameters were kept constant throughout the NPs’ production. The studied dependent variables were the size of the NPs (Y_1_), PDI (Y_2_), zeta potential (Y_3_), and GA EE (Y_4_).

ANOVA statistical analysis demonstrated that the regression was statistically significant for all the studied responses (*p* < 0.05). Tables S2–S5 (supplementary file) present this statistical analysis. In addition, regression equations were established for each response after determining the regression coefficients (RCs), reflecting the impact of each independent variable on the response under study. The equations (Equations (S1)–(S4)) were constructed only by considering the statistically significant terms (*p* < 0.05) and are provided in the supplementary file. Two- and three-dimensional response surface plots representing the impact of each parameter on a given response in the form of a color gradient are presented in the supplementary file (Figures S1–S4). 

#### 3.1.1. Effect of the Experimental Variables on the NPs’ Size

The size of the prepared NPs ranged between 113.0 nm (formulation 15) and 371.8 nm (formulation 19) (Table S1 in the supplementary file). From the regression equation and the response surface plots (Equation (S1) and Figure S1, supplementary file), it can be inferred that all the evaluated experimental variables have a negative effect on the NPs’ size, which means that when increasing these variables, the size of the NPs decreases. Although some works state that there is a positive relation between PLGA and NPs’ size, in this work, we verified that the size of NPs only shows a linear correlation with increasing polymer concentration up to 20 mg/mL as already reported by Huang and Zhang [30]. As observed in this work, above the concentration of 20 mg/mL the sizes were similar up to almost 35 mg/mL, and for higher PLGA concentrations, the NPs’ size decreased, which is supported by the previously mentioned work. Up to a specific concentration, the increase in PLGA leads to an increase in the organic phase’s viscosity, hampering the diffusion of the solvent into the aqueous phase, thus originating emulsion droplets with larger dimensions [30]. Furthermore, increasing the percentage of PVA decreases the interfacial tension between the oil droplets and the continuous aqueous phase, forming smaller NPs [31]. Additionally, as reported by other authors, longer sonication time/more sonication cycles generate higher sonication energy, leading to rapid and uniform dispersion of the polymeric organic phase and lower polydispersity, culminating in a smaller size of the NPs [32]. 

#### 3.1.2. Effect of the Experimental Variables on the NPs’ PDI

The obtained PDI values varied between 0.026 (nanoformulation 12) and 0.333 (nanoformulation 17) (Table S2 in the supplementary file). According to the regression equation and the response surface plots (Equation (S2) and Figure S2, supplementary file), it was observed that the amount of PLGA and PVA negatively affected the response. A higher amount of polymer can promote the formation of more monodisperse samples, leading to lower PDI values [33]. Furthermore, increasing the percentage of PVA can promote the stability of the colloidal systems, generating a more homogenous suspension [34]. Contrarywise, the number of FA molecules per NP and the number of sonication cycles positively affected the NPs’ PDI. Increasing the time of sonication promotes emulsion instability by increasing the turbulence and pressure, leading to agglomeration and melting of the nanodrugs in the dispersed phase, which generates populations of different sizes [35]. 

#### 3.1.3. Effect of the Experimental Variables on the NPs’ Zeta Potential

The obtained zeta potential ranged between −23.4 mV (nanoformulation 8) and −6.6 mV (nanoformulation 1) (Table S3 in the supplementary file). From the regression equation and the response surface plots (Equation (S3) and Figure S3, supplementary file), the amount of PLGA negatively affected the studied response due to negatively charged carboxyl groups. Conversely, all the remaining experimental variables positively affected the response. As both folic acid and PVA exhibit a neutral charge, increasing these variables leads to less negative zeta potential values. Furthermore, increasing the sonication time/number of sonication cycles results in smaller particles with better size distribution. Consequently, smaller particles have a greater surface area and higher negative charge [36].

#### 3.1.4. Effect of the Experimental Variables on the GA Encapsulation

The obtained GA EE values varied between 13.0% (formulation 9) and 62.2% (formulation 15) (Table S4 in the supplementary file). According to the regression equation and the response surface plots (Equation (S4) and Figure S4, supplementary file), all experimental parameters positively affect the response variable. A larger amount of PLGA is reflected in a larger polymeric matrix, thus obtaining more space to accommodate more GA molecules. Additionally, increasing the polymer concentration increases the viscosity of the organic phase, providing greater resistance to mass transfer, thus preventing drug diffusion towards the outer aqueous phase, resulting in a higher EE [37]. Furthermore, using the optimal PVA concentration also affects the diffusion of the drug at the water/oil interface [38]. Moreover, increasing the number of sonication cycles/time is reflected in faster evaporation of the oil phase, causing a quicker hardening of the PLGA. Thus, the diffusion of the drug in the aqueous phase is hindered, increasing the EE [39]. 

### 3.2. Physicochemical Properties of Optimized NPs

After the validation of the experimental design, optimized FA-GA-PLGA NPs were prepared. The criteria for obtaining the optimal formulation were as follows: for the size of the NPs, a range of values between 140 and 200 nm was established (Y_1_), the PDI value was set to less than 0.09 (Y_2_), the range of zeta potential values was maintained without any restriction, since all obtained nanoformulations were considered acceptable (Y_3_), and the EE of GA was maximized up to 100% (Y_4_). Accordingly, the optimal values of the independent variables predicted by the software were 40 mg of PLGA, 4% (*w*/*v*) PVA, 398 molecules of FA per NP, and four sonication cycles.

Then, five replicas of the checkpoint formulation with the optimal characteristics were produced. Table 2 compares the optimal responses predicted by the model and the obtained experimental results.

Table 2 shows that all the obtained responses of the prepared nanoformulations fall within the software-predicted range, revealing an excellent predictive value and mathematical adjustment of the model. The EE values are adequate corresponding to 1.3 ± 0.1% of loading capacity (the ratio of the encapsulated drug to the polymer mass). Additionally, the obtained optimal nanoformulation exhibited ideal characteristics for the brain tumor delivery of GA. Particle size is crucial for the internalization process of the drug in the target tissue. Thus, particles smaller than 200 nm are ideal for nano-delivery since they can accumulate in the tumor tissue effectively due to the enhanced permeability and retention (EPR) effect [40]. Additionally, sizes higher than 100 nm may reduce the possibility of rapid elimination from the body. The obtained formulation presented PDI values below 0.1, indicating the monodispersity of the sample.

**Figure 1:**
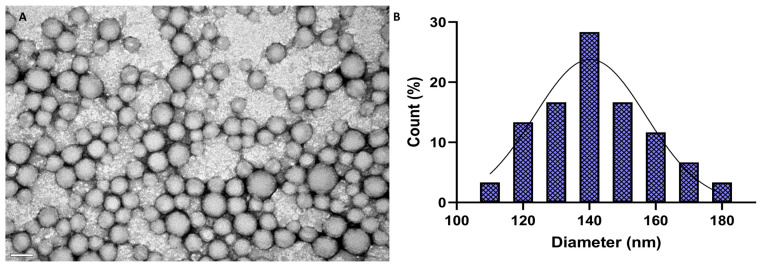
(**A**) Morphological analysis of FA-GA-loaded PLGA NPs by TEM. The scale bar corresponds to 200 nm. (**B**) Histogram of the sizes measured by TEM with a Gaussian distribution fitting (Graphpadprism, version 9.1.2).

Furthermore, the NPs exhibited highly negative zeta potential values due to the negative charge of the PLGA’s carboxylic groups. Although some evidence indicates that neutral or cationic NPs can better penetrate the BBB, negatively charged NPs are usually less toxic than cationic NPs. Positively charged NPs can present toxicity because they can interact with cell membranes, potentially leading to cell damage and inflammatory responses. Additionally, cationic NPs can strongly bind to the negatively charged DNA, causing damage to it [41]. Also, neutral-charged NPs can exhibit less stability compared to negatively charged NPs [42], which can aggregate and lose their effectiveness over time. Thus, the highly negative zeta potential values of the NPs suggest an excellent stability of the colloidal system. In fact, the NPs proved to be stable under storage conditions (aqueous suspension, 4 °C) for at least 8 weeks (Table S6 in the supplementary file). Besides the electrostatic stabilization, the colloidal stability can also be attributed to the steric effect of the PVA. This biocompatible polymer promotes colloidal stabilization through its adsorption on the NPs’ surface, thus avoiding the agglomeration of the nanoformulations [43]. Additionally, the TEM analysis (Figure 1) revealed that the NPs present a spherical and regular shape, as expected for polymeric NPs. It was also verified that the mean size determined by TEM was smaller (140 ± 17 nm) than the one obtained by DLS, as the latter measures the hydrodynamic size of the NPs, constituted by the nanoparticle–electron ion layer assembly [44]. Furthermore, the size distribution histogram obtained from the TEM analysis revealed NPs with diameters ranging from 110 to 180 nm, complying with the Gaussian distribution. 

To further confirm the success of the NPs’ conjugation with FA molecules, FTIR analysis was performed. FA was chosen as the targeting moiety for an active targeting strategy since its receptor is reportedly overexpressed in both the BBB [45] and GBM cells [46]. The conjugation was achieved by the chemical bond between the amine group of FA and the carboxyl group of PLGA mediated by EDC. EDC is one of the most widely used carbodiimides to catalyze bond formation between the carboxylic and amine groups [47].

**Figure 2:**
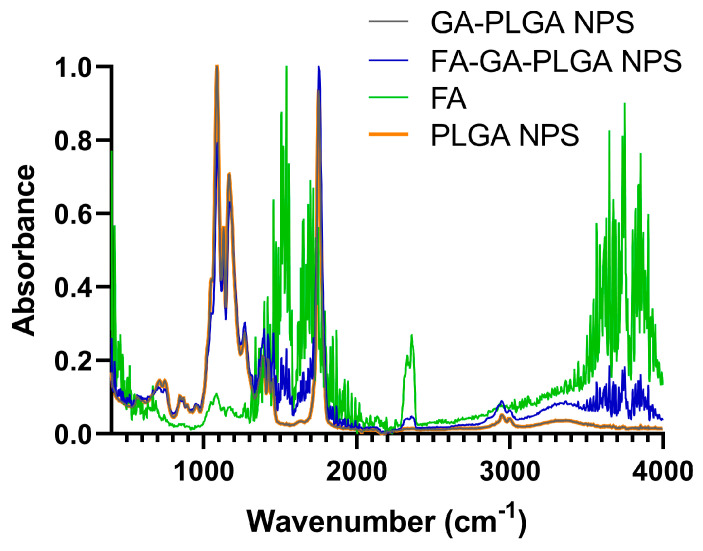
FTIR absorbance spectrum of FA-GA-PLGA NPs, GA-PLGA NPs, PLGA NPs, and FA stock solution.

Figure 2 shows that both non-modified and FA-modified PLGA NPs exhibit the PLGA characteristic peaks. The PLGA spectrum (the spectra with peak identification can also be found in Figure S5A in the supplementary file) can be divided into five main regions: 3450–3500 cm^−1^, corresponding to OH end group; 2885–3010 cm^−1^, corresponding to C-O stretching; around 1762.6 cm^−1^, due to C=O stretching; between 1450 and 850 cm^−1^, due to C-H bends; and finally, 1186–1089 cm^−1^, due to the C-O stretching [48]. 

Moreover, as depicted in Figure 2, after conjugation with FA, it is possible to see that the FA-GA-PLGA NPs present characteristic peaks of this compound, thus revealing the confirmation of this functionalization. In the FA absorption spectrum (Figure S5B in the supplementary file), it is possible to find the following: 3100–3500 cm^−1^, corresponding to OH carboxylic of glutamic acid moiety and the NH group of pterin ring stretching; 1693 cm^−1^, corresponding to the C=O carboxylic group; 1604–1605 cm^−1^, for N-H bending; and 1485–1519 cm^−1^, corresponding to phenyl and pterin ring. There are also other peaks such as 2362 cm^−1^, which is attributed to the N+-H stretching vibration band of C=N+-H- on the PT ring; 1650 cm^−1^ for C=O amide; 1639 cm^−1^, corresponding to C=N, and 1619 cm^−1^, related to C=C aromatic [49,50].

### 3.3. GA Release Profile of the Optimized NPs

**Figure 3:**
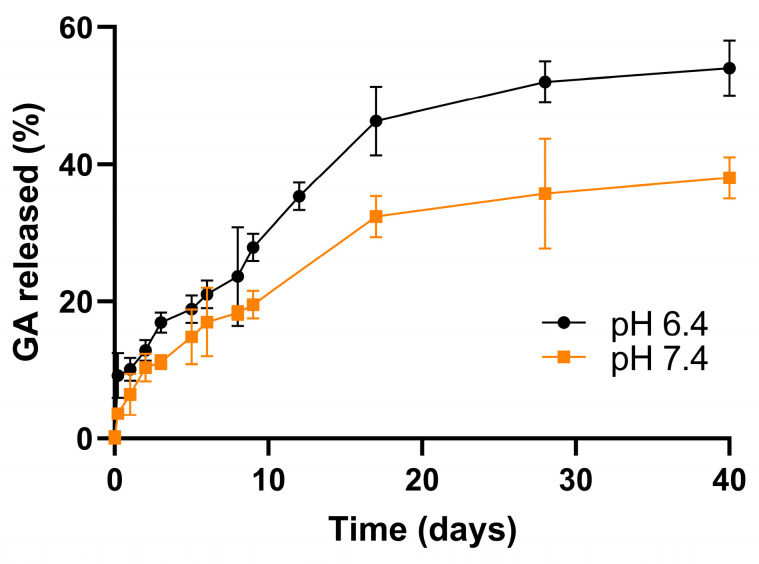
Release profile of FA-GA-loaded PLGA NPs over 40 days for pH = 7.4 and 6.4.

The GA release from the optimized nanoformulations was evaluated over time (37 °C in PBS, 0.01 M). PBS is an isotonic buffer widely used in biological applications, as it can mimic the human body’s pH, osmolarity, and ionic concentrations and shows no toxicity [51]. In this work, two different physiological conditions were simulated by varying the pH of the release media. A pH of 6.4 was chosen to simulate the tumor acidic environment since the pH in tumor tissues is reported to be between 6.2 and 6.8 [52]. To simulate the blood circulation and healthy tissues, a pH of 7.4 was employed [53]. Figure 3 shows the obtained release profiles of GA under the two studied physiological conditions.

As observed in Figure 3, in the first 48 h, an initial burst release was observed due to the de-adsorption of GA molecules adsorbed to the NPs’ surface [54], with about 10 ± 2 and 13 ± 1% of GA being released at pH 7.4 and 6.4, respectively. After this initial burst release, the compound was released in a slower and more sustained manner. This slower release can occur by three different pathways: (i) drug diffusion from the polymeric matrix, (ii) surface hydrolysis, and (iii) bulk erosion [55]. The stability of the NPs under the two simulated physiological conditions was evaluated to understand the pathway involved in the GA release. It was verified that the physicochemical properties remained constant during the experiment (Table S7 in the supplementary file), suggesting that the drug release occurs mainly by drug diffusion and not due to the erosion of the polymeric matrix. 

It is reported that the release profile depends also on the chemical properties of the encapsulated molecule (e.g., solubility and diffusion) and the features of the release medium, such as its pH [56]. In fact, the obtained results revealed that the pH of the media influenced the GA release profile. For example, it was observed that after 40 days, while at pH 6.4 the NPs released 54 ± 4% of GA, at pH 7.4 the NPs only released 38 ± 3% of GA (*p* < 0.05). The observed higher release at a lower pH can be justified by the fact that GA is an ionizable compound. With increasing pH, GA suffers ionization due to the deprotonation of its carboxy- and hydroxy-groups, which affects its solubility in the release buffer [57]. 

Thus, the obtained results revealed that the NPs are an efficient carrier for the delivery of GA to brain tumors since these can selectively release more compound in the acidic tumor environment, promoting its accumulation in the target cells while decreasing the amount of compound loss in the blood circulation and healthy tissues. Furthermore, the achieved slow and controlled release is advantageous to minimize the off-target toxicity associated with high initial doses. Thus, the developed NPs are suitable for maintaining consistent therapeutic levels of GA, while they may also offer a favorable safety profile. 

### 3.4. NPs’ Uptake by Endocytosis Mediated by the FA Receptor

**Figure 4:**
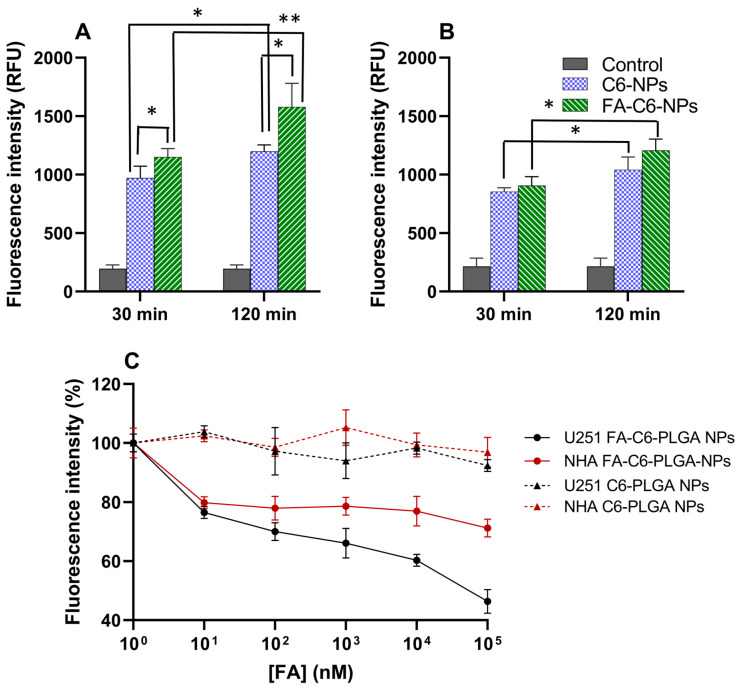
Fluorescence quantification of coumarin-6-labeled NPs’ uptake by (**A**) U251 and (**B**) NHA cells after two incubation periods (30 and 120 min). The control corresponds to the non-treated cells’ autofluorescence. (**C**) Effect of the FA receptor blockage on the NPs’ uptake. The cells were pre-treated with excess FA for 1 h before 2 h of incubation with NPs. * *p* < 0.05 and ** *p* < 0.01 indicate a statistically significant difference between groups.

The NPs’ uptake was quantified by fluorescence measurements in a GBM human cell line (U251) and healthy immortalized human astrocytes (NHA) as control. To allow for NPs’ quantification, the NPs were labeled with the green-fluorescent dye coumarin-6. The cells were treated with non-modified and FA-modified NPs for 30 and 120 min to evaluate the effect of FA conjugation and the incubation period. The obtained results are presented in Figure 4A,B. 

Figure 4A,B revealed that the NPs’ uptake is time-dependent, with longer incubation times resulting in increased NPs’ internalization. For example, in U251 cells, increasing the incubation time from 30 to 120 min enhanced the uptake of FA-conjugated and non-conjugated NPs by about 37% and 23%, respectively (*p* < 0.05). Additionally, it was observed that the FA conjugation promoted the NPs’ uptake in both cell lines. However, this effect was more evident in the GBM cells than healthy astrocytes. After 2 h of incubation, while for U251 cells, the FA conjugation enhanced cell internalization by about 32%, for NHA cells, only a 16% increase was verified (*p* < 0.05). The effect of FA conjugation was also less pronounced for shorter periods of incubation, since after 30 min, the uptake of FA-NPs in U251 cells only increased by 18% compared with non-conjugated NPs. This may be justified by lower incubation times, giving less time for the NPs to be recognized and consequently bind to the FA receptor.

The observed higher uptake of the FA-NPs in the GBM cells than in the healthy astrocytes may suggest that the NPs’ internalization occurs by endocytosis mediated by the FA receptor. 

Further studies were conducted to confirm this by blocking the FA receptor. The cells were pre-treated with increasing concentrations of FA before incubating with the NPs. The obtained results are presented in Figure 4C. As the results show, pretreatment with excess FA decreased the NPs’ uptake; however, this effect was more pronounced in the tumor cells than in healthy astrocytes. While for U251 cells, a FA concentration of 1.0 × 10^5^ nM inhibited the uptake of FA-conjugated NPs by about 54%, only a 29% decrease was observed for NHA cells (*p* < 0.05). These results are justified by the overexpression of FA receptors in the tumor cells [58]. Furthermore, blocking the FA receptor did not significantly affect the uptake of non-modified NPs in both cell lines (*p* > 0.05), where the maximal FA concentration only decreased the NPs’ internalization by 8 and 3% in U251 and NHA cells, respectively. Altogether, the obtained results confirm that FA conjugation promotes the receptor-mediated endocytosis of the NPs and that the developed NPs are efficient carriers for the GA intracellular delivery. Other authors have provided evidence that FA-modified PLGA NPs are effectively internalized by folate receptor-mediated endocytosis, and these have provided information regarding the intracellular localization of these NPs following uptake. For example, Wang et al. (2020) observed the uptake and intracellular trafficking of FA-conjugated PLGA NPs in breast cancer cells using confocal laser scanning microscopy. The experiments demonstrated that these NPs are initially localized within endosomes and lysosomes before escaping into the cytoplasm, where they can deliver therapeutic agents effectively [59].

### 3.5. GA Encapsulation in FA-PLGA NPs Potentiate Its In Vitro Antiproliferative Activity 

**Figure 5:**
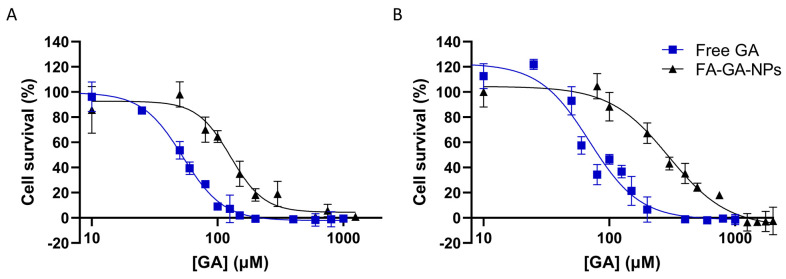
Cell survival inhibition curves of GA, free or entrapped in FA-PLGA NPs, determined by SRB assay after 72 h treatment on (**A**) human GBM cells (U251) and (**B**) human healthy astrocytes (NHA). Cell survival is presented as a percentage (% = T/C × 100). Data are given as mean ± SD (n = 3).

A human GBM cell line (U251) and healthy brain cells (NHA) were used to evaluate the in vitro antiproliferative activity of FA-GA-loaded PLGA NPs compared to free GA. The cells were treated with increasing concentrations of free GA ranging between 10 and 1000 µM and FA-GA-loaded PLGA NPs between 10 and 2000 µM, and the obtained survival inhibition curves for each cell line are presented in Figure 5. 

As Figure 5 depicts, cell survival decreases with increasing GA concentration in both cell lines regardless of whether GA is in its free form or loaded in the FA-modified NPs. Furthermore, it appears that free GA possesses a higher antiproliferative effect for all tested concentrations than entrapped GA, with the survival rates being practically null at free GA concentrations above 200 µM. This observation is also consistent with the determined half-maximal inhibitory concentration values in Table 3 below. 

As depicted by Table 3, the FA-GA-PLGA NPs’ IC50 values are higher than the ones obtained for free GA in both cell lines (*p* < 0.05), suggesting that FA-GA-PLGA NPs have a lower antiproliferative activity than free GA. However, this can be explained by the slow release of GA from the FA-PLGA NPs, resulting in an inability to produce the same intensity of antiproliferative activity as the free compound, as previously reported by other authors [11]. The release experiments revealed that after the cell treatment period (72 h), only approximately 16.9% of GA was released from the FA-PLGA NPs in tumor acidic conditions. Considering that the total concentration of added GA is not available after that period, for a more realistic comparison with the effect of free GA, estimated IC50 values for the FA-GA-PLGA NPs were determined considering the amount of GA that would have been released after 72 h (16.9%) instead of the total GA entrapped in the NPs. Table 3 presents the estimated IC50 values between parentheses. For both U251 and NHA cells, the estimated IC50 values are significantly lower than the ones for free GA (*p* < 0.05), suggesting that the NPs can promote compound antitumoral effect. 

**Figure 6:**
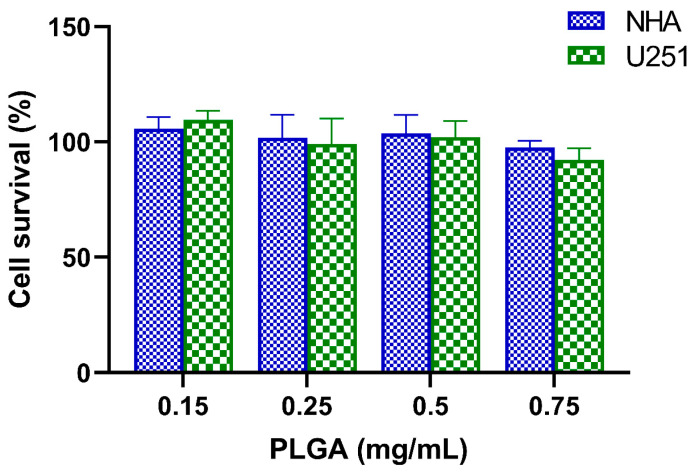
Effect of control unloaded FA-PLGA NPs on the cell survival of U251 and NHA cells, determined by SRB assay after 72 h treatment. Cell survival is presented as a percentage (% = T/C × 100). Data are given as mean ± SD (n = 3). The physicochemical characterization of these NPs can be found in Table S8 in the Supplementary Material.

Furthermore, the obtained IC50 values revealed that free GA is less toxic for the healthy cells than for the tumor cells (*p* < 0.05), as already reported [60]. Therefore, due to its lower toxicity to healthy tissues, GA can be potentially used for GBM therapy. However, since it still exhibits adverse effects towards healthy cells, NPs for its target delivery are required for an effective treatment without systemic toxicity. In fact, the obtained results showed that GA encapsulation in FA-modified NPs is advantageous to decrease toxicity towards healthy cells since lower IC50 values were obtained for the U251 cells when compared with the healthy astrocytes (*p* < 0.05). These promising results are in agreement with the higher uptake of FA-modified NPs by the tumor cells observed in Section 3.4, due to their overexpression of the FA receptor. The higher uptake by the tumor cells may lead to increased bioavailability and accumulation of the natural compound in the cell cytoplasm, consequently promoting therapeutic efficacy, 

Additionally, as shown in Figure 6 below, control unloaded FA-PLGA NPs proved to be biocompatible between the tested concentrations (0.15–0.75 mg/mL) since no significant effect on the survival of both U251 and NHA cells was observed. 

### 3.6. In Vitro Production of ROS

To further investigate the mechanisms behind the GA anti-GBM effect, the fluorometric assay using the DCFH-DA probe was employed to detect oxidative species. The non-fluorescent DCFH-DA probe is converted into a highly fluorescent molecule—2′,7′-dichlorofluorescein (DCF)—when oxidized by intracellular oxidative species, therefore allowing us to study the in vitro generation of ROS.

**Figure 7:**
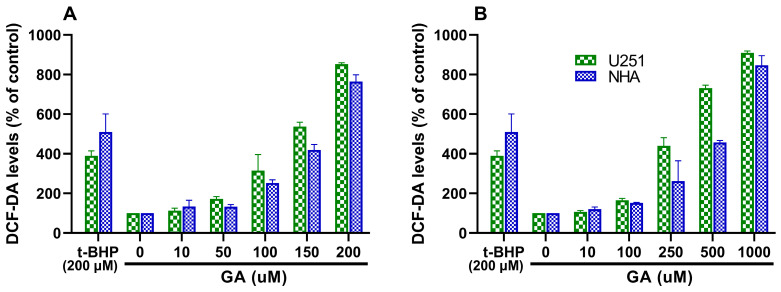
Effects of increasing concentrations of GA in the free form (**A**) or (**B**) entrapped in FA-PLGA NPs on ROS levels quantified by the DCF-DA probe in both U251 and NHA cells after 72 h treatment with increasing GA concentrations. t-BHP at a concentration of 200 µM was used as a positive control. Graphs indicate DCF (ROS) levels (%) as compared with each negative control cell (non-treated cells).

U251 and NHA cells were treated with increasing concentrations of free GA and FA-GA-PLGA NPs, and the ROS production was quantified after treatment. The obtained results are presented in Figure 7.

Figure 7 shows that ROS production increases with increasing GA concentration, either in the free form (Figure 7A) or entrapped in the NPs (Figure 7B) for both cell lines. The results also revealed that GA’s ability to induce ROS production is more prominent in the GBM cells than in the healthy astrocytes, particularly for GA concentrations above 50 and 250 µM for free GA and FA-GA-NPs, respectively (*p* < 0.05). This is in agreement with several works that have reported the ability of GA to induct ROS production in tumor cells [61,62]. 

In these experiments, t-BHP, a strong oxidant, was used as a positive control because U251 GBM cells are reported to be highly sensitive to this agent, exhibiting high intracellular peroxide production in its presence [63]. It was observed that for the tumor cells (U251), free GA at concentrations above 150 µM could surpass the ROS-production ability of 200 µM t-BHP. However, in NHA cells, to achieve a higher effect than 200 µM t-BHP, cells required free GA at concentrations above 200 µM. These results are in agreement with the cytotoxicity studies that revealed a lower toxicity of GA towards the healthy cells. Furthermore, the entrapment of GA in the FA-PLGA NPs also proved to help protect healthy tissues from the deleterious effects of GA, since to exceed the effect of 200 µM t-BHP, it required higher concentrations of FA-GA-NPs for NHA cells than for tumor cells (250 vs. 1000 µM).

Additionally, it can be verified that to achieve the same level of ROS production, apparently a higher dose of FA-GA-PLGA NPs is required than free GA, which can also be justified by the slow release rates as already mentioned. It is important to note that since the total amount of entrapped GA is not available by the end of the experiment (72 h), this does not mean definitively that the NPs are less efficient compared to the free compound. For example, for U251 cells, 200 µM of free GA induced the production of 852 ± 7% of DCF as compared with control cells, while 1000 µM of GA-loaded NPs induced the production of 910 ± 9% of DCF. However, if we consider the 16.9% release of GA from the NPs, it is possible to estimate that the effective concentration of GA released is about 169 µM. Thus, suggesting that a lower concentration of released GA produced a higher effect than the free compound and that GA encapsulation in the developed NPs does not affect the mechanism behind cell death induction. 

## 4. Conclusions

In the last decade, nanomedicine has emerged as a major weapon in the fight against tumors. As GBM is one of the deadliest types of cancer, seeking new effective therapies is a continuous challenge. In this research, PLGA NPs were proposed to encapsulate the natural compound GA as a targeted drug delivery strategy to overcome the limitations of the current GBM therapeutic strategies mainly related to high toxicity. The surface of the developed NPs was successfully conjugated with FA, promoting the selectivity of the NPs for the target tumor GBM cells. Interestingly, at first glance, GA encapsulation in the developed NPs appeared to decrease the antitumor activity of GA. However, those results were explained due to the slow release rate. Thus, it was possible to conclude that the enhanced accumulation of the natural compound in the tumor cells led to an improved therapeutic efficacy associated with the induction of intracellular oxidative stress and lower toxicity to the healthy cells. Therefore, the compound encapsulation in FA-conjugated PLGA NPs may offer a novel approach to circumvent the systemic toxicity associated with the current GBM therapy. 

## Figures and Tables

**Table 1:** Independent variables and applied levels in the experimental design.

Parameters	Component	Units	Applied Level				
			−α	−1	0	+1	+α
A	m_PLGA_	mg	1.8	10	25	40	48.2
B	PVA	%	0.18	1	2.5	4	4.82
C	FA molecules/NP	Unit	18	100	250	400	482
D	Sonication cycles	Unit	1	2	4	6	7

Note: m_PLGA_—PLGA mass; PVA—percentage of PVA; FA molecules/NP—number of folic acid molecules per nanoparticle; sonication cycles—number of sonication cycles of 10 s each.

**Table 2:** Predicted versus experimental values. The experimental results are represented as mean ± SD (n = 5).

	Predicted Values	Experimental Values
Size (nm)	140 (123–157)	153 ± 9 (142–163)
PDI	0.05 (0.02–0.07)	0.05 ± 0.01 (0.03–0.06)
Zeta Potential (mV)	−21.9 (−32.7–[−18.9])	−27 ± 2 (−25.4–[−29.5])
GA EE (%)	56 (48–64)	51 ± 4 (46–55)

**Table 3:** IC_50_ values for GA determined after 72 h treatment with the free drug or entrapped in FA-PLGA NPs. Data are given as mean ± SD (n = 3). Between parentheses are given the IC5_0_ values estimated based on GA released at the 72 h timepoint, considering a 16.9% release observed in Figure 3.

IC_50_ (µM)		
	Free GA	FA-GA-PLGA NPs
U251	48.7 ± 6.0	136.6 ± 29.1 (23.1 ± 4.9)
NHA	87.2 ± 7.4	277.5 ± 22.3 (47.0 ± 3.8)

## Data Availability

The original contributions presented in the study are included in the article, further inquiries can be directed to the corresponding author.

## Supplementary Materials

The following supporting information can be downloaded at: https://www.mdpi.com/article/10.3390/polym16152161/s1, Table S1: Experimental design and obtained responses of all 30 nanoformulations; Table S2: ANOVA analysis for the response variable size (Y_1_); Table S3: ANOVA analysis for the response variable PDI (Y_2_); Table S4: ANOVA analysis for the response variable zeta potential (Y_3_); Table S5: ANOVA analysis for the response variable GA EE (Y_4_); Table S6: Optimal FA-GA-loaded PLGA NPs’ stability in storage conditions over 8 weeks (mean ± SD. n = 5); Table S7: Optimal FA-GA-loaded PLGA NPs’ stability in physiological conditions over 40 days (mean ± SD. n = 3); Table S8: Physicochemical properties of the control unloaded FA-conjugated PLGA NPs used for the biocompatibility studies.; Equations (S1)–(S4): Regression Equations for NP size; Figure S1: (A,B) Contour and (C,D) surface plots showing the effect of the different independent variables on the NPs’ size; Figure S2: (A,B) Contour and (C,D) surface plots showing the effect of the different independent variables on the NPs’ PDI; Figure S3: (A,B) Contour and (C,D) surface plots showing the effect of the different independent variables on the NPs’ zeta potential; Figure S4: (A,B) Contour and (C,D) surface plots showing the effect of the different independent variables on the NPs’ EE of GA; Figure S5: FTIR absorbance spectra of (A) PLGA NPs and (B) stock FA.
