# Supplementary material for: Folic-Acid-Conjugated Poly (Lactic-Co-Glycolic Acid) Nanoparticles Loaded with Gallic Acid Induce Glioblastoma Cell Death by Reactive-Oxygen-Species-Induced Stress
Source: Polymers (Basel). 2024 Jul 30;16(15):2161. doi: 10.3390/polym16152161 (PMC11313823; doi:10.3390/polym16152161)
Supplement: Supplementary file 1 [file polymers-16-02161-s001.zip › polymers-3112256-supplementary.pdf]

Supplementary Information

Folic acid-conjugated PLGA nanoparticles loaded with Gallic acid induce glioblastoma cells death by ROS-induced stress

Maria João Ramalho [1,2,] *#, Bruna Alves [1,2] #, Stephanie Andrade [1,2], Jorge Lima [3,4,5], Joana Angélica Loureiro [1,2] and Maria Carmo Pereira [1,2] *

[1] LEPABE - Laboratory for Process Engineering, Environment, Biotechnology and Energy, Faculty of Engineering, University of Porto, Rua Dr. Roberto Frias, 4200-465 Porto, Portugal

[2] ALiCE - Associate Laboratory in Chemical Engineering, Faculty of Engineering, University of Porto, Rua Dr. Roberto Frias, 4200-465 Porto, Portugal

[3] i3S - Instituto de Investigação e Inovação em Saúde, Universidade do Porto, R. Alfredo Allen, 4200-10 135 Porto, Portugal

[4] Ipatimup - Instituto de Patologia e Imunologia Molecular da Universidade do Porto, Rua Júlio Amaral de Carvalho 45, 4200-135, Porto, Portugal

[5] Faculty of Medicine of Porto University, Alameda Prof. Hernâni Monteiro, 4200-319, Porto, Portugal

* Corresponding authors: mjramalho@fe.up.pt; mcsp@fe.up.pt

A - Experimental design

Table S1. Experimental design and obtained responses of all 30 nanoformulations. The experimental levels (low, center, high, and alpha) are represented by -1, 0, +1, and α, respectively.

Run order	Coded independent variables							
	A	B	C	D	Mean diameter (nm)	PDI	Zeta Potential (mV)	EE GA (%)
**1**	-1	-1	-1	-1	158.3	0.039	-6.6	21.6
**2**	+1	-1	-1	-1	181.1	0.036	-10.3	34.8
**3**	-1	+1	-1	-1	181.0	0.052	-20.5	47.0
**4**	+1	+1	-1	-1	158.4	0.028	-22.9	47.0
**5**	-1	-1	+1	-1	158.3	0.065	-15.1	23.3
**6**	+1	-1	+1	-1	195.6	0.05	-18.5	28.1
**7**	-1	+1	+1	-1	143.8	0.063	-13.3	57.4
**8**	+1	+1	+1	-1	153.9	0.051	-23.4	44.4
**9**	-1	-1	-1	+1	143.9	0.056	-7.1	13.0
**10**	+1	-1	-1	+1	174.1	0.041	-8.9	34.5
**11**	-1	+1	-1	+1	130.4	0.081	-15.5	47.4
**12**	+1	+1	-1	+1	135.2	0.026	-20.5	55.1
**13**	-1	-1	+1	+1	167.7	0.062	-13.4	17.9
**14**	+1	-1	+1	+1	193.8	0.117	-19.6	16.8
**15**	-1	+1	+1	+1	113.0	0.062	-12.6	62.2
**16**	+1	+1	+1	+1	121.0	0.048	-20.6	50.6
**17**	- α	0	0	0	193.1	0.333	-19.4	48.8
**18**	+ α	0	0	0	163.6	0.088	-21.2	54.2
**19**	0	- α	0	0	371.8	0.115	-23.3	24.7
**20**	0	+ α	0	0	140.0	0.098	-18.9	62.0
**21**	0	0	- α	0	145.2	0.057	-21.4	44.2
**22**	0	0	+ α	0	139.8	0.058	-19.9	56.3
**23**	0	0	0	- α	171.5	0.028	-22.0	41.4
**24**	0	0	0	+ α	143.0	0.075	-19.1	60.1
**25**	0	0	0	0	154.9	0.034	-19.6	43.2
**26**	0	0	0	0	141.9	0.061	-17.5	27.5
**27**	0	0	0	0	140.3	0.053	-22.2	28.1
**28**	0	0	0	0	155.2	0.043	-19.0	40.7
**29**	0	0	0	0	160.0	0.037	-19.5	46.1
**30**	0	0	0	0	156.4	0.036	-19.4	48.8

Table S2. ANOVA analysis for the response variable Size (Y_1).

			**Size (Y_1)**			
Source	Sum of Squares	df	Mean Square	F-value	p-value	
**Model**	56949.58	24	2372.90	34.99	0.0004	significant
A-PLGA	435.13	1	435.13	6.42	0.0523	
B-PVA	26865.62	1	26865.62	396.14	< 0.0001	
C-FA	14.58	1	14.58	0.2150	0.6624	
D-Sonication	406.13	1	406.13	5.99	0.0581	
AB	842.45	1	842.45	12.42	0.0168	
AC	133.98	1	133.98	1.98	0.2188	
AD	28.89	1	28.89	0.4260	0.5428	
BC	1077.48	1	1077.48	15.89	0.0105	
BD	956.36	1	956.36	14.10	0.0132	
CD	95.55	1	95.55	1.41	0.2886	
A^2	1085.42	1	1085.42	16.00	0.0103	
B^2	16364.70	1	16364.70	241.30	< 0.0001	
C^2	120.15	1	120.15	1.77	0.2406	
D^2	50.46	1	50.46	0.7440	0.4278	
ABC	40.64	1	40.64	0.5993	0.4739	
ABD	52.93	1	52.93	0.7804	0.4175	
ACD	144.60	1	144.60	2.13	0.2041	
BCD	22.33	1	22.33	0.3292	0.5910	
A^2B	13337.89	1	13337.89	196.67	< 0.0001	
A^2C	2.30	1	2.30	0.0338	0.8613	
A^2D	0.2177	1	0.2177	0.0032	0.9570	
AB^2	1043.28	1	1043.28	15.38	0.0112	
ABCD	7.43	1	7.43	0.1095	0.7541	
A^2B^2	4767.22	1	4767.22	70.29	0.0004	
Pure Error	339.09	5	67.82			
**Cor Total**	57288.68	29				

Table S3. ANOVA analysis for the response variable PDI (Y_2).

PDI (Y_2)						
Source	Sum of Squares	df	Mean Square	F-value	p-value	
**Model**	0.0890	24	0.0037	31.76	0.0006	significant
A-PLGA	0.0300	1	0.0300	256.96	< 0.0001	
B-PVA	0.0001	1	0.0001	1.24	0.3166	
C-FA	5.000E-07	1	5.000E-07	0.0043	0.9504	
D-Sonication	0.0011	1	0.0011	9.46	0.0276	
AB	0.0010	1	0.0010	8.63	0.0323	
AC	0.0008	1	0.0008	6.59	0.0502	
AD	0.0000	1	0.0000	0.3344	0.5881	
BC	0.0005	1	0.0005	3.87	0.1064	
BD	0.0002	1	0.0002	2.12	0.2048	
CD	7.563E-06	1	7.563E-06	0.0647	0.8093	
A^2	0.0416	1	0.0416	356.02	< 0.0001	
B^2	0.0059	1	0.0059	50.17	0.0009	
C^2	0.0003	1	0.0003	2.34	0.1866	
D^2	0.0001	1	0.0001	0.7224	0.4342	
ABC	1.562E-06	1	1.562E-06	0.0134	0.9124	
ABD	0.0005	1	0.0005	4.43	0.0892	
ACD	0.0008	1	0.0008	6.59	0.0502	
BCD	0.0003	1	0.0003	2.85	0.1521	
A^2B	0.0000	1	0.0000	0.1336	0.7297	
A^2C	0.0003	1	0.0003	2.91	0.1485	
A^2D	0.0003	1	0.0003	2.22	0.1968	
AB^2	0.0202	1	0.0202	172.74	< 0.0001	
ABCD	0.0002	1	0.0002	1.50	0.2748	
A^2B^2	0.0180	1	0.0180	154.24	< 0.0001	
Pure Error	0.0006	5	0.0001			
**Cor Total**	0.0896	29				

Table S4. ANOVA analysis for the response variable Zeta Potential (Y_3).

Zeta (Y_3)						
Source	Sum of Squares	df	Mean Square	F-value	p-value	
**Model**	638.18	24	26.59	11.42	0.0065	significant
A-PLGA	1.62	1	1.62	0.6955	0.4423	
B-PVA	9.95	1	9.95	4.27	0.0937	
C-FA	1.08	1	1.08	0.4639	0.5261	
D-Sonication	4.12	1	4.12	1.77	0.2410	
AB	6.88	1	6.88	2.95	0.1464	
AC	13.52	1	13.52	5.81	0.0609	
AD	0.0977	1	0.0977	0.0419	0.8458	
BC	117.34	1	117.34	50.38	0.0009	
BD	5.65	1	5.65	2.43	0.1800	
CD	1.02	1	1.02	0.4358	0.5383	
A^2	0.8702	1	0.8702	0.3736	0.5678	
B^2	3.66	1	3.66	1.57	0.2655	
C^2	1.90	1	1.90	0.8175	0.4074	
D^2	1.58	1	1.58	0.6788	0.4475	
ABC	2.90	1	2.90	1.24	0.3153	
ABD	0.0086	1	0.0086	0.0037	0.9540	
ACD	0.0001	1	0.0001	0.0000	0.9963	
BCD	0.8327	1	0.8327	0.3575	0.5759	
A^2B	76.29	1	76.29	32.75	0.0023	
A^2C	14.34	1	14.34	6.16	0.0557	
A^2D	0.0853	1	0.0853	0.0366	0.8558	
AB^2	14.11	1	14.11	6.06	0.0571	
ABCD	5.63	1	5.63	2.42	0.1808	
A^2B^2	70.21	1	70.21	30.15	0.0027	
Pure Error	11.65	5	2.33			
**Cor Total**	649.83	29				

Table S5. ANOVA analysis for the response variable GA EE (Y_4).

GA EE (Y_4)						
Source	Sum of Squares	df	Mean Square	F-value	p-value	
**Model**	3824.88	4	956.22	11.17	<0.0001	significant
A-PLGA Mass	42.88	1	42.88	0.5008	0.4857	
B-%PVA	3739.55	1	3739.55	43.68	<0.0001	
C-Fa molecules/NP	17.40	1	17.40	0.2032	0.6560	
D-Sonication Cycles	25.06	1	25.06	0.2927	0.5933	
**Residual**	2140.25	25	85.61			
Lack of Fit	1722.24	20	86.11	1.03	0.5409	not significant
Pure Error	418.01	5	83.60			
**Cor Total**	5965.14	29				

Equation for NPs Size

Size = 151.45 -9.54 (PLGA mass) – 74.93 * (%PVA) – 1.75 (FA molecules/NP) - 9.21 (Sonication Cycles) – 7.26 (PLGA mass) (%PVA) - 8.21 (%PVA) (FA molecules/NP) - 7.73 (%PVA) (Sonication Cycles) + 11.24 $(\text{PLGA mass})^2$ + 43.66 $(\%\text{PVA})^2$ + 60.18 $(\text{PLGA mass})^2$ (%PVA) + 16.83 (PLGA mass) $(\%\text{PVA})^2$ - 48.19 $(\text{PLGA mass})^2$ $(\%\text{PVA})^2$ (Eq. S1)

Equation for NPs PDI

PDI = 0.0440 – 0.0792 (PLGA mass) – 0.0055 (%PVA)
+ 0.0003 (FA molecules/NP) + 0.0152 (Sonication Cycles)
– 0.0079 (PLGA mass) (%PVA) + 0.0696 $(\text{PLGA mass})^2$
+ 0.0261 (%PVA) + 0.0740 (PLGA mass) $(\%\text{PVA})^2$
- 0.0937 $(\text{PLGA mass})^2$ $(\%\text{PVA})^2$ (Eq. S2)

Equation for NPs Zeta Potential

Zeta Potential = -19.54 -0.5819 (PLGA mass) + 1.44 (%PVA)
+ 0.4752 (FA molecules/NP) + 0.9278 (Sonication Cycles)
- 7.26 (%PVA) (FA molecules/NP)
- 8.21 $(\text{PLGA mass})^2$ (%PVA) – 7.73 $(\text{PLGA mass})^2$ $(\%\text{PVA})^2$ (Eq. S3)

Equation for NPs EE

GA EE = 40.91 + 1.44 (PLGA mass) + 13.41 (%PVA)
+ 0.9149 (FA molecules/NP) + 1.10 (Sonication Cycles) (Eq. S4)

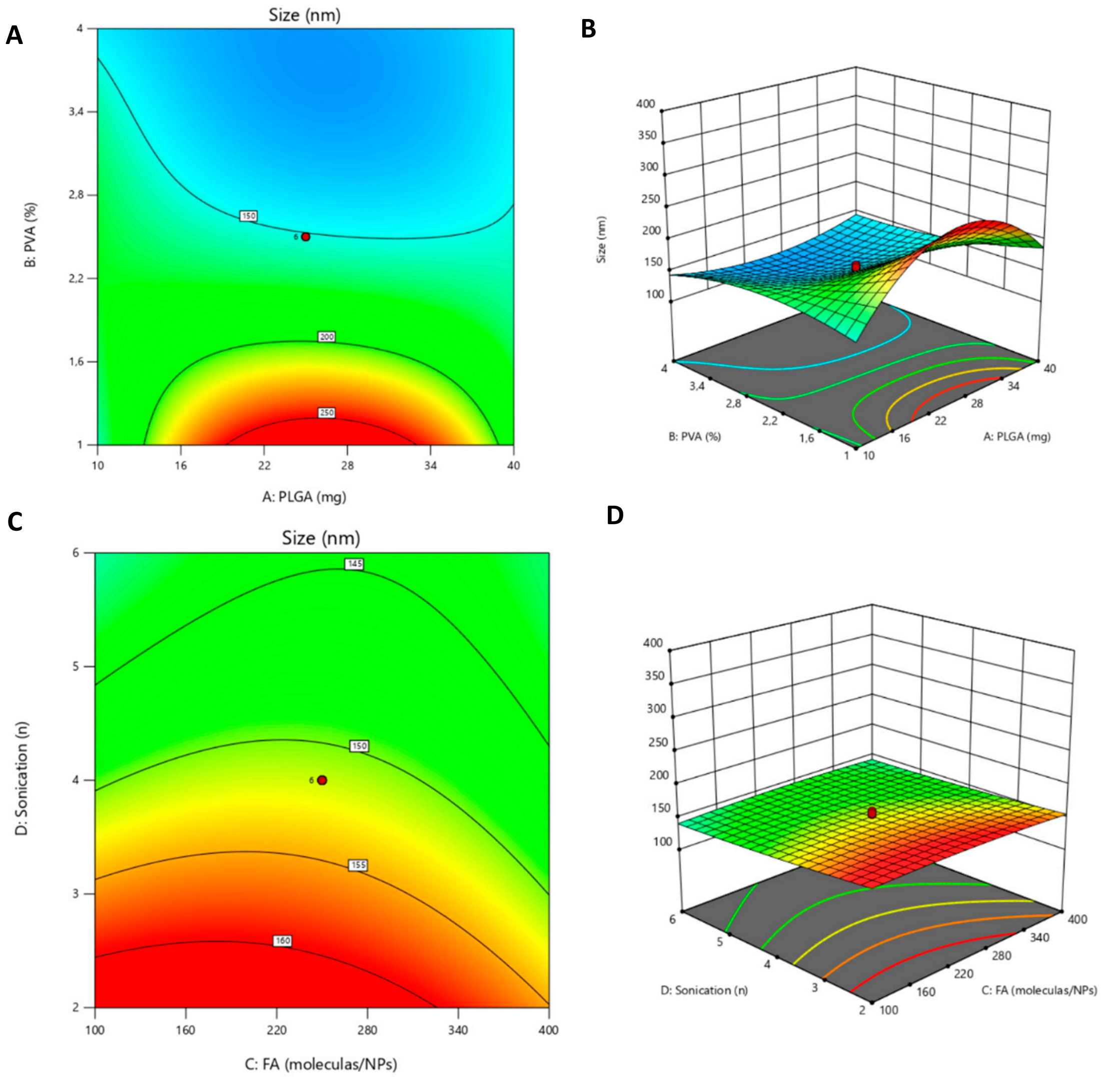

Figure S1. (A)(B) Contour and (C)(D) surface plots showing the effect of the different independent variables on the NPs' size.

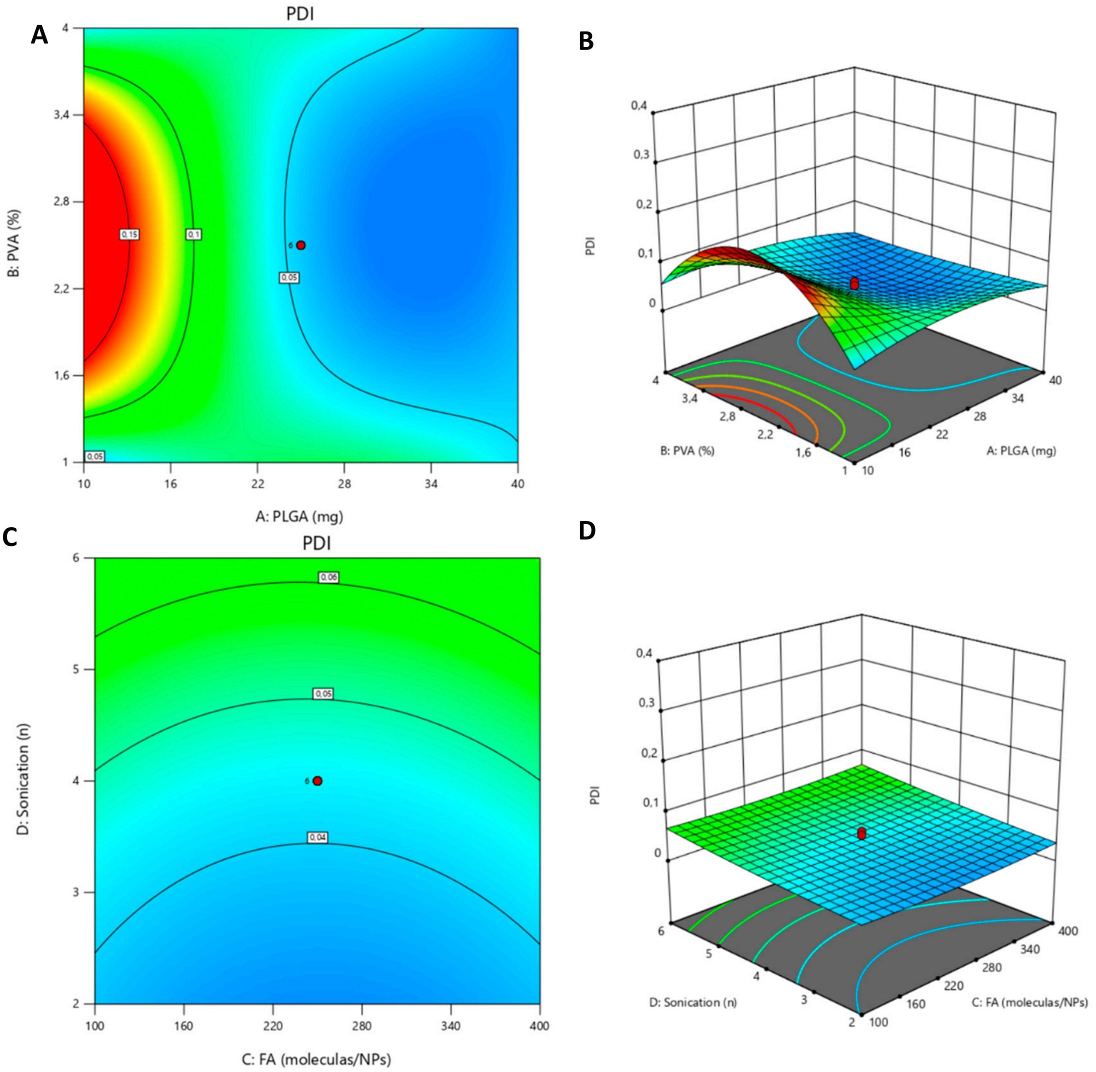

Figure S2. (A)(B) Contour and (C)(D) surface plots showing the effect of the different independent variables on the NPs' PDI.

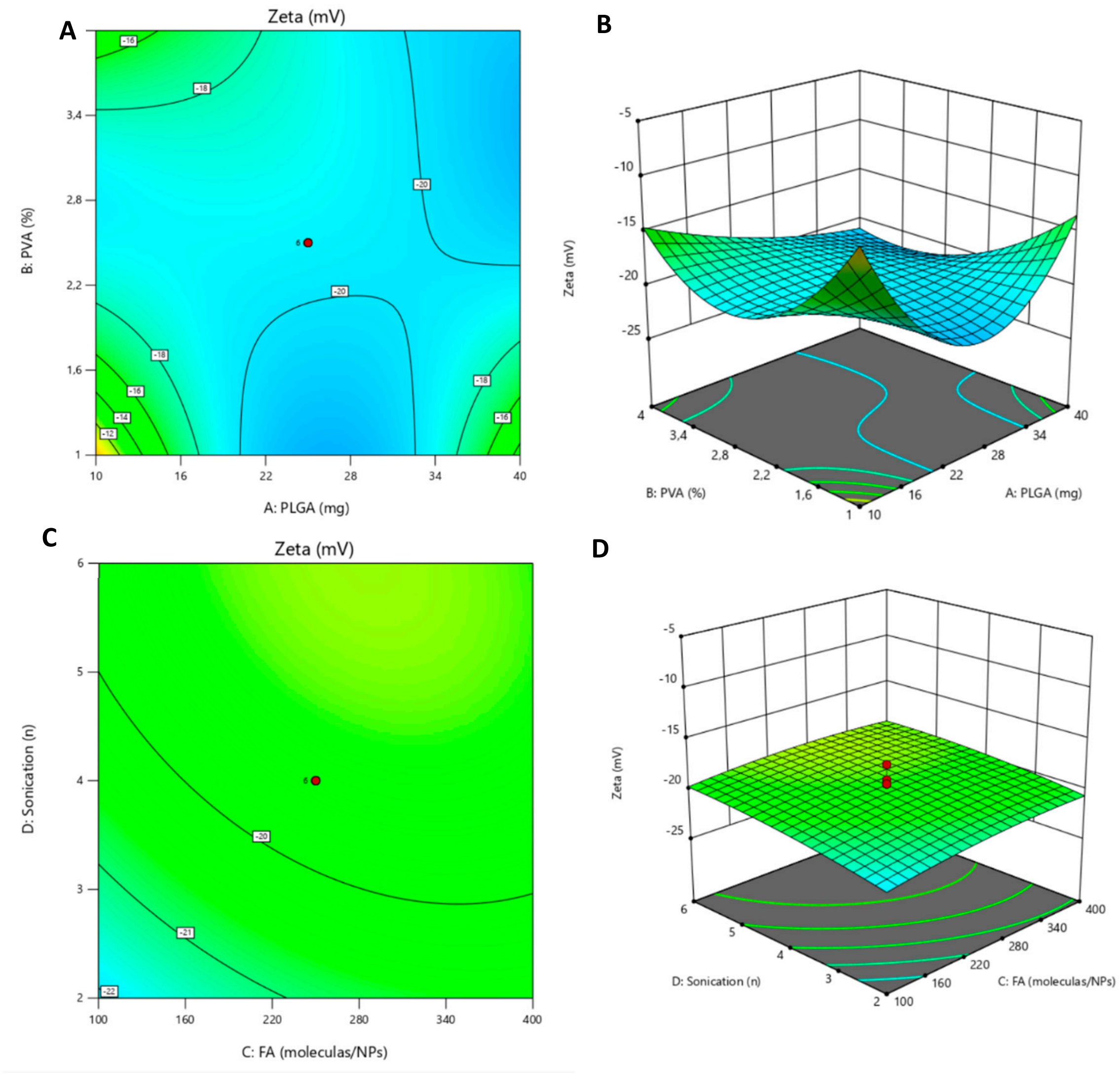

Figure S3. (A)(B) Contour and (C)(D) surface plots showing the effect of the different independent variables on the NPs' zeta potential.

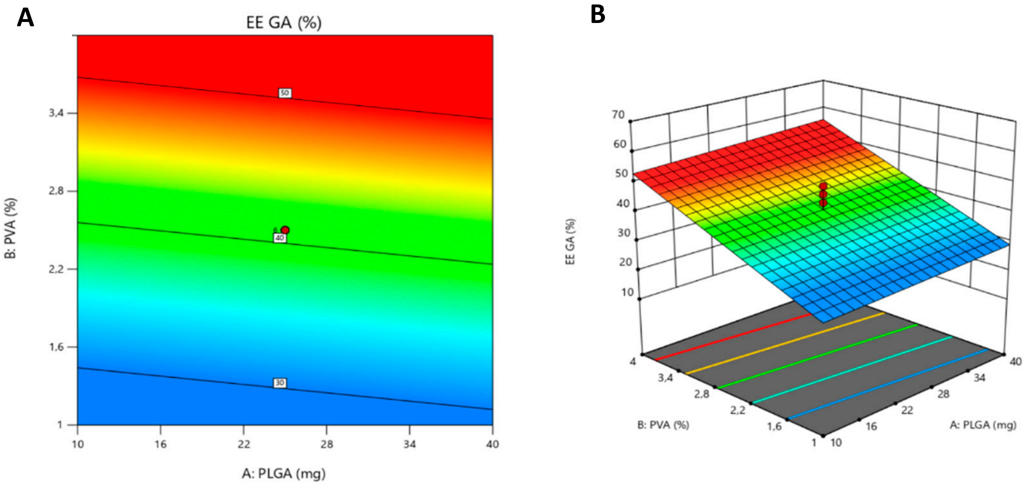

Figure S4. (A) Contour and (B) surface plots of PLGA mass and % of PVA on the NPs' EE of GA.

B- NPs' colloidal stability

Table S6. Optimal FA-GA-loaded PLGA NPs stability in storage conditions over 8 weeks (mean ± SD. n=5). The NPs were stored in aqueous suspension at 4 ºC, and their stability was measured in terms of changes in size, PDI and zeta potential using the DLS technique once a week. No statistically significant changes were observed in the NPs' size and PDI values ($p>0.05$). A decrease in the zeta potential values was observed ($p<0.05$), however this change does not reflect in the NPs instability, since it may occur due to successively applying an electric potential in the sample during the DLS measurements.

Time (weeks)	Mean size (nm)	PDI	Zeta potential (mV)
**0**	153 ± 9	0.05 ± 0.01	- 27 ± 2
**1**	153 ± 9	0.03 ± 0.01	- 22 ± 3
**2**	150 ± 9	0.040 ± 0.005	- 21 ± 2
**3**	152 ± 9	0.06 ± 0.03	- 21 ± 2
**4**	150 ± 8	0.04 ± 0.01	- 22 ± 1
**5**	152 ± 7	0.04 ± 0.01	- 22 ± 1
**6**	151 ± 8	0.05 ± 0.01	- 21 ± 2
**7**	152 ± 8	0.04 ± 0.02	- 23 ± 2
**8**	149 ± 9	0.04 ± 0.01	- 20 ± 2

Table S7. Optimal FA-GA-loaded PLGA NPs stability in physiological conditions over 40 days (mean ± SD. n=3). The NPs were kept in simulated physiological conditions (37 ºC, gentle agitation, PBS 0.01 M, pH 6.4 or pH 7.4), and their stability was measured in terms of changes in size, PDI and zeta potential using the DLS technique. As observed, the zeta potential values are very close to neutral due to the PBS's charge neutralization effect. The free charges in the PBS interact with the carboxyl groups on the surface of the NPs, masking the negative charge of the NPs.

Time (days)	Mean size (nm)	PDI	Zeta potential (mV)
**PBS 0.01 M, pH 6.4**			
**0**	152 ± 4	0.02 ±	-1.8 ±0.1
**1**	145 ± 15	0.05 ± 0.02	-1.2 ± 0.01
**2**	140 ± 9	0.04 ± 0.01	-2.5 ± 0.2
**3**	150 ± 2	0.02 ±	-2.5 ± 1.7
**6**	144 ± 15	0.04 ± 0.01	-1.7 ± 1.1
**9**	150 ± 6	0.06 ± 0.03	-3.1 ± 1.0
**12**	143 ± 5	0.02 ±	-4.3 ± 2.1
**17**	146 ± 16	0.05 ± 0.01	-2.5 ± 0.1
**28**	140 ± 8	0.05 ± 0.03	-7.4 ± 0.3
**40**	143 ± 3	0.08 ± 0.02	-6.2 ± 0.2
**PBS 0.01 M, pH 7.4**			
**0**	147 ± 5	0.03 ± 0.01	-1.4 ± 0.5
**1**	144 ± 5	0.04 ± 0.01	-1.7 ± 0.5

**2**	144 ± 1	0.02 ± 0.01	-1.2 ± 0.3
**3**	148 ± 1	0.03 ± 0.01	-2.7 ± 0.3
**6**	152 ± 15	0.06 ± 0.01	-0.7 ± 0.3
**9**	141 ± 18	0.06 ± 0.04	-3.0 ± 0.01
**12**	139 ± 3	0.03 ± 0.02	-4.3 ± 1.2
**17**	145 ± 8	0.06 ± 0.01	-1.7 ± 0.1
**28**	141 ± 7	0.02 ± 0.03	- 4.5 ± 0.4
**40**	146 ± 5	0.07 ± 0.04	- 3.9 ± 1.1

C - FTIR analysis

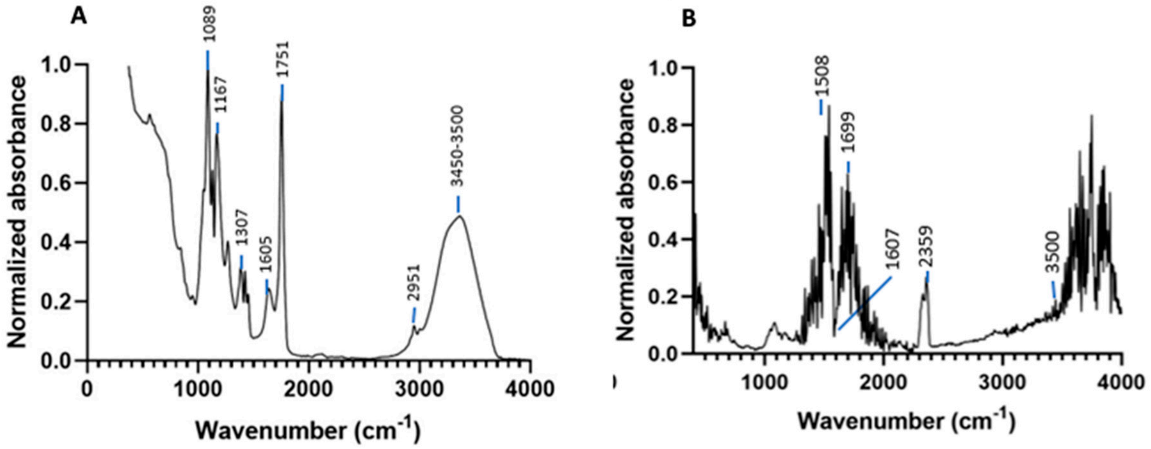

Figure S5. FTIR absorbance spectra of (A) PLGA NPs and (B) Stock FA.

D - NPs' biocompatibility studies

Table S8. Physicochemical properties of the control unloaded FA-conjugated PLGA NPs used for the biocompatibility studies.

**Size (nm)**	**PDI**	**Zeta potential (mV)**
146 ± 4	0.06 ± 0.03	- 23.2 ± 3.4
